# Supplementary material for: Resolving the full spectrum of human genome variation using Linked-Reads
Source: Genome Res. 2019 Apr;29(4):635–45. doi: 10.1101/gr.234443.118 (PMC6442396; doi:10.1101/gr.234443.118)
Supplement: Supplemental Material [file supp_gr.234443.118_Supplemental_longranger-2.2.zip › longranger-2.2.tar.bz2!/lr2.2-public/lib/stan/cmdstan/stan/doc/api/html/multinomial_8hpp_source.html]

Stan: src/stan/prob/distributions/multivariate/discrete/multinomial.hpp Source File


|  |  |
| --- | --- |
|  | Stan  2.5.0  probability, sampling & optimization |


- Main Page
- Related Pages
- Namespaces
- Classes
- Files

- File List
- File Members

All Classes Namespaces Files Functions Variables Typedefs Enumerations Enumerator Friends Macros Pages

- src
- stan
- prob
- distributions
- multivariate
- discrete

multinomial.hpp

Go to the documentation of this file.

1 #ifndef STAN\_\_PROB\_\_DISTRIBUTIONS\_\_MULTIVARIATE\_\_DISCRETE\_\_MULTINOMIAL\_HPP

2 #define STAN\_\_PROB\_\_DISTRIBUTIONS\_\_MULTIVARIATE\_\_DISCRETE\_\_MULTINOMIAL\_HPP

3

4 #include <boost/math/special\_functions/gamma.hpp>

5 #include <boost/random/uniform\_01.hpp>

6 #include <boost/random/variate\_generator.hpp>

7

8 #include <stan/math/error\_handling.hpp>

9 #include <stan/math/error\_handling.hpp>

10 #include <stan/math/functions/multiply\_log.hpp>

11 #include <stan/math/matrix\_error\_handling.hpp>

12 #include <stan/prob/constants.hpp>

13 #include <stan/prob/distributions/univariate/discrete/binomial.hpp>

14 #include <stan/prob/distributions/multivariate/discrete/categorical.hpp>

15 #include <stan/prob/traits.hpp>

16

17 namespace stan {

18

19  namespace prob {

20  // Multinomial(ns|N,theta) [0 <= n <= N; SUM ns = N;

21  // 0 <= theta[n] <= 1; SUM theta = 1]

22  template <bool propto,

23  typename T\_prob>

24  typename boost::math::tools::promote\_args<T\_prob>::type

25  multinomial\_log(const std::vector<int>& ns,

26  const Eigen::Matrix<T\_prob,Eigen::Dynamic,1>& theta) {

27  static const char\* function = "stan::prob::multinomial\_log(%1%)";

28

29  using stan::math::check\_nonnegative;

30  using stan::math::check\_simplex;

31  using stan::math::check\_size\_match;

32  using boost::math::tools::promote\_args;

33  using boost::math::lgamma;

34

35  typename promote\_args<T\_prob>::type lp(0.0);

36  check\_nonnegative(function, ns, "Number of trials variable", &lp);

37  check\_simplex(function, theta, "Probabilites parameter",

38  &lp);

39  check\_size\_match(function,

40  ns.size(), "Size of number of trials variable",

41  theta.rows(), "rows of probabilities parameter",

42  &lp);

43  using stan::math::multiply\_log;

44

45  if (include\_summand<propto>::value) {

46  double sum = 1.0;

47  for (unsigned int i = 0; i < ns.size(); ++i)

48  sum += ns[i];

49  lp += lgamma(sum);

50  for (unsigned int i = 0; i < ns.size(); ++i)

51  lp -= lgamma(ns[i] + 1.0);

52  }

53  if (include\_summand<propto,T\_prob>::value)

54  for (unsigned int i = 0; i < ns.size(); ++i)

55  lp += multiply\_log(ns[i], theta[i]);

56  return lp;

57  }

58

59  template <typename T\_prob>

60  typename boost::math::tools::promote\_args<T\_prob>::type

61  multinomial\_log(const std::vector<int>& ns,

62  const Eigen::Matrix<T\_prob,Eigen::Dynamic,1>& theta) {

63  return multinomial\_log<false>(ns,theta);

64  }

65

66  template <class RNG>

67  inline std::vector<int>

68  multinomial\_rng(const Eigen::Matrix<double,Eigen::Dynamic,1>& theta,

69  const int N,

70  RNG& rng) {

71  static const char\* function = "stan::prob::multinomial\_rng(%1%)";

72  using stan::math::check\_simplex;

73  using stan::math::check\_positive;

74

75  check\_simplex(function, theta, "Probabilites parameter", (double\*)0);

76  check\_positive(function,N,"number of trials variables", (double\*)0);

77

78  std::vector<int> result(theta.size(),0);

79  double mass\_left = 1.0;

80  int n\_left = N;

81  for (int k = 0; n\_left > 0 && k < theta.size(); ++k) {

82  double p = theta[k] / mass\_left;

83  if (p > 1.0) p = 1.0;

84  result[k] = binomial\_rng(n\_left,p,rng);

85  n\_left -= result[k];

86  mass\_left -= theta[k];

87  }

88  return result;

89  }

90

91

92  }

93 }

94 #endif

multiply\_log.hpp

traits.hpp

stan::math::check\_size\_match

bool check\_size\_match(const char \*function, T\_size1 i, const char \*name\_i, T\_size2 j, const char \*name\_j, T\_result \*result)

**Definition:** check\_size\_match.hpp:13

error\_handling.hpp

stan::math::multiply\_log

boost::math::tools::promote\_args< T\_a, T\_b >::type multiply\_log(const T\_a a, const T\_b b)

Calculated the value of the first argument times log of the second argument while behaving properly w...

**Definition:** multiply\_log.hpp:51

stan::math::check\_simplex

bool check\_simplex(const char \*function, const Eigen::Matrix< T\_prob, Eigen::Dynamic, 1 > &theta, const char \*name, T\_result \*result)

Return true if the specified vector is simplex.

**Definition:** check\_simplex.hpp:32

stan::prob::multinomial\_rng

std::vector< int > multinomial\_rng(const Eigen::Matrix< double, Eigen::Dynamic, 1 > &theta, const int N, RNG &rng)

**Definition:** multinomial.hpp:68

matrix\_error\_handling.hpp

constants.hpp

stan::agrad::sum

fvar< T > sum(const Eigen::Matrix< fvar< T >, R, C > &m)

**Definition:** sum.hpp:14

stan::agrad::lgamma

fvar< T > lgamma(const fvar< T > &x)

**Definition:** lgamma.hpp:15

stan::prob::binomial\_rng

int binomial\_rng(const int N, const double theta, RNG &rng)

**Definition:** binomial.hpp:470

stan::prob::include\_summand

Template metaprogram to calculate whether a summand needs to be included in a proportional (log) prob...

**Definition:** traits.hpp:35

binomial.hpp

stan::math::check\_nonnegative

bool check\_nonnegative(const char \*function, const T\_y &y, const char \*name, T\_result \*result)

**Definition:** check\_nonnegative.hpp:52

stan::math::check\_positive

bool check\_positive(const char \*function, const T\_y &y, const char \*name, T\_result \*result)

**Definition:** check\_positive.hpp:54

stan::agrad::multiply\_log

fvar< T > multiply\_log(const fvar< T > &x1, const fvar< T > &x2)

**Definition:** multiply\_log.hpp:15

stan::prob::multinomial\_log

boost::math::tools::promote\_args< T\_prob >::type multinomial\_log(const std::vector< int > &ns, const Eigen::Matrix< T\_prob, Eigen::Dynamic, 1 > &theta)

**Definition:** multinomial.hpp:25

categorical.hpp


---

[ Stan Home Page ]

*© 2011–2014,
Stan Development Team.*
